# Seed defense biopriming with bacterial cyclodipeptides triggers immunity in cucumber and pepper

**DOI:** 10.1038/s41598-017-14155-9

**Published:** 2017-10-27

**Authors:** Geun Cheol Song, Hye Kyung Choi, Young Sook Kim, Jung Sup Choi, Choong-Min Ryu

**Affiliations:** 1 Molecular Phytobacteriology Laboratory, KRIBB, Daejeon, 34141 South Korea; 2 Biosystems and Bioengineering Program, University of Science and Technology, Daejeon, 34113 South Korea; 3 Eco-Friendly New Materials Research Center, KRICT, Daejeon, 34114 South Korea

## Abstract

Seed priming is to expose seeds to specific compounds to enhance seed germination. Few studies of plant immune activation through seed priming have been conducted. Here, we introduce an emerging technology that combines seed priming with elicitation of plant immunity using biologically active compounds. This technology is named ‘seed defense biopriming’ (SDB). We prepared heat-stable metabolites from 1,825 root-associated *Bacillus* spp. isolated from the rhizosphere in South Korea. These preparations were tested for their ability to induce SDB in cucumber and pepper seeds and trigger plant immunity. SDB with heat-stable metabolites of the selected *Bacillus gaemokensis* strain PB69 significantly reduced subsequent bacterial diseases under *in vitro* and field conditions and increased fruit yield. Transcriptional analysis of induced resistance marker genes confirmed the upregulation of salicylic acid, ethylene, and jasmonic acid signaling. Mortality of the insect pest *Spodoptera litura* increased when larvae fed on SDB-treated cucumber tissues. Analysis of the causative bacterial metabolites identified a leucine-proline cyclodipeptide and a commercially obtained leucine-proline cyclodipeptide induced similar results as treatment with the bacterial preparation. Our results indicate that SDB treatment with the heat-stable bacterial metabolite effectively elicited immunity and controlled disease in seedlings to whole plants, thereby increasing yield even under field conditions.

## Introduction

Crop protection from insect pests and pathogens has gained paramount significance for food security worldwide^1^. Chemical pesticides are used to protect crops from pathogens, but these chemicals have serious adverse effects such as toxicity for humans and livestock, ecosystem disruption, and environmental pollution^1^. Biopesticides may provide an alternative approach to crop protection, and include a number of control methods derived from natural substances or bioproducts. Some studies have investigated how to select, produce, and apply biopesticides^2^. Biopesticide application methods include foliage spray, soil drenching, and seed treatment. Seed treatment is particularly promising because it should enable seedlings to mount a robust immune response, and thereby remain disease-free (or only moderately infected) for a long time with minimal labor and expense. Previous studies focused on seed treatment applications to protect plants from soil-borne diseases, which usually affect seeds before germination and are difficult to manage^2,3^. Another benefit of seed treatment with biopesticides is that it synchronizes seed priming with seed germination^4^. Seed priming refers to the use of natural or synthetic compounds to induce a particular physiological state in seedlings before germination^5^. Many strategies other than biopesticides have been used for seed priming, including hydropriming, osmopriming, chemical priming, hormonal priming, biological priming, redox priming, and solid matrix priming^6,7^. However, few studies have examined plant immune activation through seed priming.

Plants mount an array of inducible resistance responses to herbivores, pathogens, and chemicals via *de novo* production of toxic metabolites, which reduce or inhibit further attacks^8^. Inducible resistance is regulated primarily by three phytohormones, salicylic acid (SA), jasmonic acid (JA), and ethylene (ET), which are interconnected by complex signaling networks and crosstalk phenomena^9^. There are two kinds of inducible defense responses that depend on the elicitor, induced systemic resistance (ISR) and systemic acquired resistance (SAR)^10^. ISR is elicited in response to plant growth-promoting rhizobacteria (PGPR), and is mediated primarily by JA and ET signaling pathways. SAR is elicited in response to chemical triggers and a wide range of necrotizing pathogens^9,11–13^, and is mediated by SA signaling^8,14,15^. Generally, JA-mediated ISR responses are directed against herbivores and necrotrophic pathogens, whereas SA-mediated SAR responses are directed against biotrophic pathogens^16,17^. SAR occurs after the hypersensitive response (HR), which is a highly specific interaction between a plant resistance protein and a pathogenic avirulent protein leading to programmed cell death and pathogen growth arrest in infected plant tissue^18–21^. ISR does not require HR.

Induced defense responses such as SAR are generally linked with allocation costs in the form of reduced growth and reproduction^22^. Benzothiadiazole (BTH) is a classic example of a chemical trigger used to elicit SAR that inflicts a growth penalty^23^. This phenomenon is called ‘allocation fitness cost’ or ‘trade-off’^24^. Growth reduction is attributed to the competing demands between metabolic biosynthetic pathways and the energy required for induced defense responses^25^. However, some elicitors for other induced defense responses such as ISR are not associated with allocation fitness costs. The bacterial volatile compounds (e.g., 2,3-butanediol and acetoin) and their derivatives (e.g., 3-pentanol and 4-aminobenzoic acid) stimulate growth and induced resistance in *Arabidopsis thaliana* and pepper, but do not incur an allocation fitness cost^26–29^. In the majority of cases, SAR usually leads immediately to an increase in the expression of defense-related genes, whereas ISR instantly induces gene expression after the plant is exposed to the pathogen. Then, the induction of defensive capacity is faster and stronger at the moment the plant senses the elicitor, which is known as ‘defense priming’^30^. This suggests that induced defense elicitors derived from beneficial bacteria like PGPR may not incur a plant growth penalty.

Worrall and colleagues recently reported that the application of JA and β-aminobutyric acid (BABA) priming agents on tomato seeds generated plants with primed defense responses^31^. Plants grown from JA-treated seeds displayed increased resistance to herbivory by spider mites, caterpillars, and aphids, and resistance to the necrotrophic fungal pathogen *Botrytis cinerea* ^24^. These results indicate that treatment of seeds with priming agents to elicit resistance enables plants to effectively mount long-term resistance responses against plant pathogens and insects. Defense priming agents must be carefully validated before use under field conditions, particularly with respect to their effects on allocation fitness cost, environmental impacts, effects of light and heat, and economic cost. For example, although JA and BABA elicit induced resistance, certain concentrations of JA and BABA negatively affect plant growth^24^. The biosafety of any chemicals used for induced resistance must be evaluated before field use, which could delay their implementation until better biosafety evaluation protocols are established.

Many bacterial metabolites may satisfy the criteria for use as an agent of plant defense priming. Plant-associated bacteria evolved in parallel with plants, and their metabolites interact with plants as herbicides, phytotoxins, plant growth regulators, defense inducers, and phytoalexins^32^. Many metabolites have been identified in *Bacillus* spp. that control plant diseases; these are known as PGPR^33^. *Bacillus* spp. synthesize a diverse array of bioactive metabolites such as surfactin, iturin, and fengycin^34^. Bacterial amphiphilic cyclic peptides (lipopeptides; LPs) protect plants via direct disease resistance and ISR^35^. Bacterial cyclodipeptides are a class of small molecules that exhibit diverse biological activities. For example, cyclo(L-Phe–D-Pro) and cyclo(L-Leu–L-Pro) act as antifungal compounds against *Fusarium sporotrichioides* and *Aspergillus parasiticus*, respectively^36,37^. These results confirm that bacterial cyclodipeptides directly kill pathogens. However, the application of bacterial cyclodipeptides to elicit defense priming or seed priming has not been studied.

In this study, we propose a new method referred to as ‘seed defense biopriming’ (SDB), which uses bacterial heat-stable metabolites to elicit induced resistance in cucumber and pepper. We verify that SDB does not incur a growth penalty. We demonstrate that cyclodipeptides isolated from *Bacillus gaemokensis* strain PB69 elicit induced resistance in cucumber and pepper. Our results indicate that SDB effectively elicits immunity from the seed to the whole plant, and enables plants to maintain effective disease control without a growth penalty under greenhouse and field conditions.

## Results

### Optimizing seed defense biopriming

**Figure 1:**
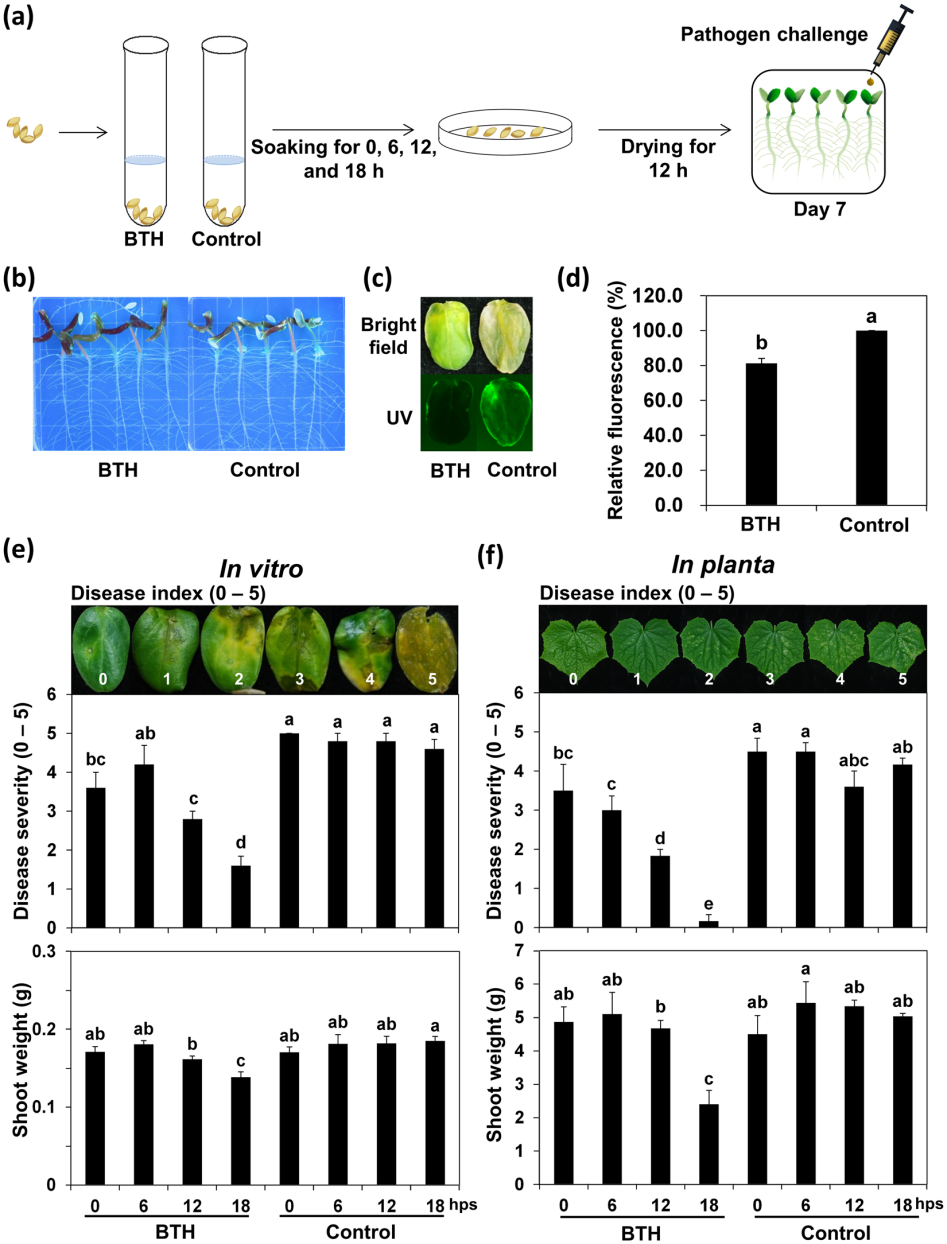
Screen to identify optimal parameters for a novel seed defense biopriming protocol in cucumber. (**a**) Schematic of the experimental design. (**b**) Representative photograph of cucumber seedlings growing in water-agar (WA) 7 days post-inoculation (dpi) with *Pseudomonas syringae* pv. lachrymans. (**c**) Representative photograph of disease symptoms taken under white light and UV irradiation. (**d**) Quantification of *P. syringae* pv. lachrymans::green fluorescence protein (GFP) fluorescence shown in (**c**). (**e**−**f**) Shoot weight and disease severity (0−5) of cucumber seedlings treated with 1 mM benzothiadiazole (BTH) were assessed 7 days after sowing and 7 days after infection with *P. syringae* pv. lachrymans *in vitro* (**e**) and *in planta* (**f**), respectively. Disease symptom severity was scored from 0 to 5 as follows. (**e**) 0, No disease symptoms; 1, yellowish color; 2, chlorosis only; 3, partial necrosis and chlorosis; 4, necrosis of the inoculated area and expanded chlorosis; and 5, complete necrosis of the inoculated area. (**f**) 0, No disease symptoms; 1, number of disease symptoms < 20; 2, number of disease symptoms < 40; 3, number of disease symptoms < 60; 4, number of disease symptoms < 80; and 5, number of disease symptoms < 100. Seeds were soaked in 1 mM BTH for 0, 6, 12, and 18 h. Water was used as the negative control. Bars represent the mean ± SE (*n* = 5 replicates per treatment). Different letters indicate significant differences between treatments (*P* = 0.05 according to least significant difference). The experiment was repeated four times with similar results.

Our aim was to investigate whether seedling defense responses could be primed by submerging seeds in autoclaved cultures of heat-resistant and endospore-forming soil bacteria, mainly *Bacillus* spp. First, we performed a proof-of-concept experiment by treating cucumber seeds with the well-characterized chemical triggers of induced resistance (Supplementary Figure S1). SDB was optimized with respect to the following parameters: (1) time that seeds were submerged; (2) BTH concentration; and (3) pathogen selection and challenge protocol. Cucumber is a model plant for testing ISR because it grows rapidly and has a well-established pathosystem (Fig. 1a). To effectively screen for SDB, we developed an *in vitro* pathosystem using *Pseudomonas syringae* pv. lachrymans tagged with GFP (Fig. 1b,c). This system has two advantages. First, one set of experiments can be completed within 14 days, which significantly reduced the previous time requirement of at least 21 days^38,39^. Second, the use of GFP fluorescence intensity as a marker for bacterial quantification enabled rapid and accurate screening compared with the standard protocol of macerating leaf tissue, culturing the extract, and counting bacterial colonies.

We evaluated the magnitude of induced resistance response and recorded any changes in plant growth resulting from induced resistance (Fig. 1). We used 1 mM BTH as a model chemical trigger of SAR to optimize the protocol parameters. The results indicated that plants grown *in vitro* exhibited the greatest disease resistance to *P. syringae* pv. lachrymans when seeds were submerged in BTH for 12 and 18 h compared with the responses in control plants (Fig. 1b–e). The shoot weight of plants generated from seeds that were treated with 1 mM BTH for 6 h did not significantly differ from that of control plants, yet they still displayed robust SDB-elicited induced resistance to protect cucumber seedlings against *P. syringae* pv. lachrymans (Fig. 1e,f). Seed treatment with 1 mM BTH for 18 h was associated with reduced plant growth. The optimum time for seed treatment was 12 h, which achieved strong defense priming, robust ISR in plants, and minimal plant growth penalty (Fig. 1e,f)^40^. These proof-of-concept experiments enabled optimization of the SDB screening methods, including seed treatment time, pathogen challenge protocol, bacterial quantification method, and parameters for minimal growth penalty.

### SDB induced by heat-stable metabolites from rhizosphere *Bacillus* spp

**Figure 2:**
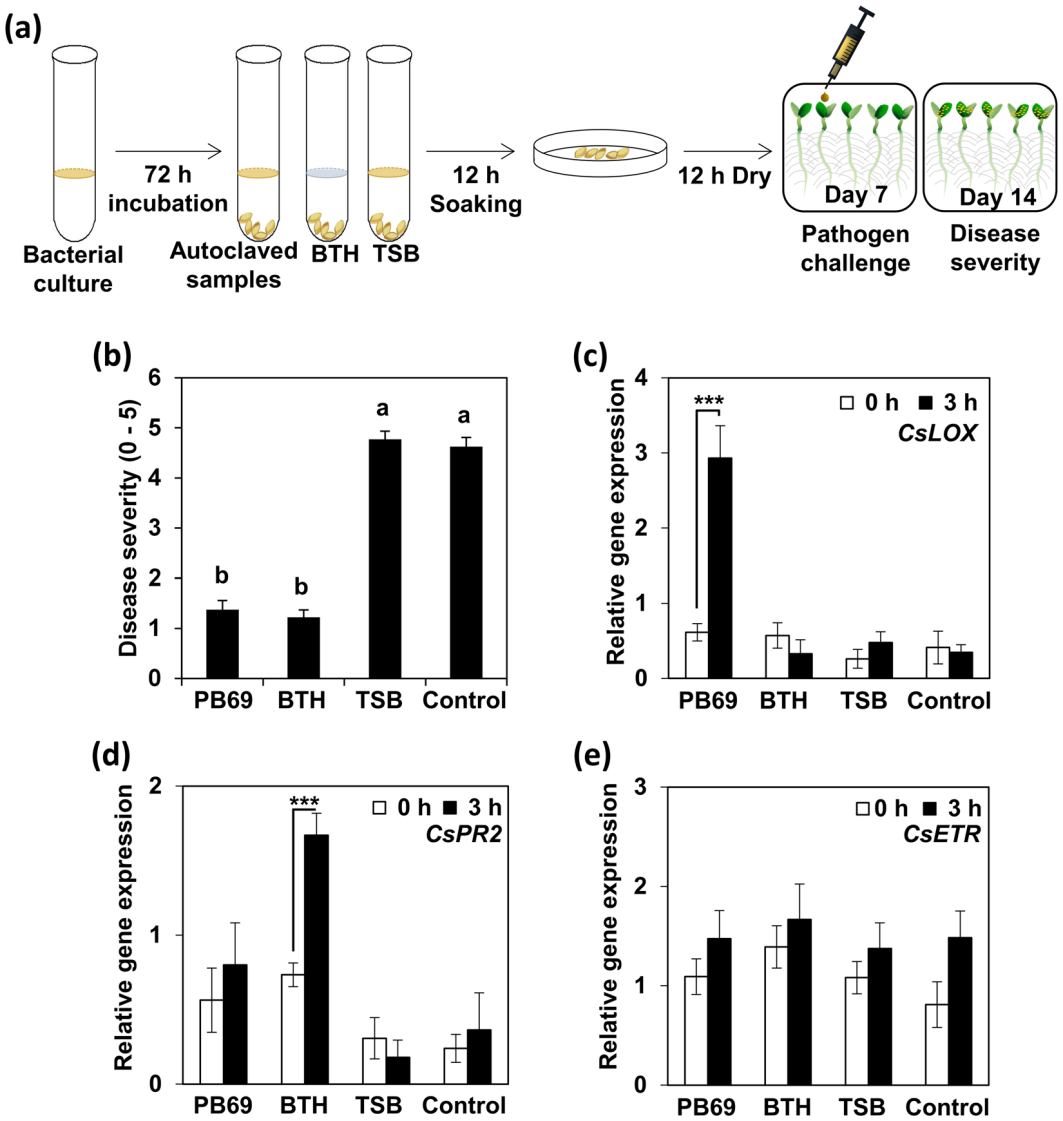
Screening of autoclaved bacterial media from 1,825 isolates for agents that elicit induced resistance in cucumber plants *in vitro*. (**a**) Schematic of the experimental design. BTH was used as a positive control for the induction of systemic acquired resistance and to select optimal protocol parameters. Seeds were submerged in 1 mM BTH for 12 h. Primed seeds were planted in square Petri dishes containing water-agar (WA). After 7 days, the *in vitro* plants were challenged with pathogen delivered to the leaf using a syringe. (**b**) Disease severity (0–5) of cucumber seedlings subjected to seed defense biopriming with autoclaved culture metabolites (ACMs) from *Bacillus gaemokensis* strain PB69 was assessed 7 days after infection with *P. syringae* pv. lachrymans. (**c**−**e**) Expression levels of the cucumber resistance genes *CsLOX* (two-way ANOVA, n = 3 experiments, ***P < 0.001, F(7,16) = 78.92), *CsPR2* (two-way ANOVA, n = 3 experiments, ***P < 0.001, F(7,16) = 13.66), and *CsETR1*(two-way ANOVA, *n* = 3 experiments, F(7,16) = 0.59) were assessed by qRT-PCR analysis at 0 and 3 h after *P. syringae* pv. lachrymans challenge in cucumber plants subjected to SDB with PB69 ACM. Bars represent the mean value ± SE (*n* = 3). Actin was used as a control. Different letters indicate significant differences between treatments (*P* = 0.05 according to least significant difference). An asterisk (***) indicates a significant difference (*P* < 0.001). The experiment was repeated twice with similar results.

A total of 1,825 *Bacillus* spp. strains were isolated from diverse plant rhizosphere and soil in S. Korea, and the bacterial cultures were used to prepare SDB tests. To rule out the effects of known *Bacillus* metabolites such as surfactin, iturins, and fengycins, the bacterial cultures were autoclaved at 121 °C for 30 min to remove bacterial cells or endospore contamination (Fig. 2a). The autoclaved cultures contained heat-stable metabolites, designated as autoclaved culture metabolites (ACMs). Seeds were treated with ACMs for 12 h, and were evaluated for induced resistance against *P. syringae* pv. lachrymans (Fig. 2a). Disease symptoms appeared after 14 days (Fig. 2a). We conducted SDB screening of the 1,825 ACMs to select an ACM that elicited similar levels of induced resistance against *P. syringae* pv. lachrymans as observed for the 1 mM BTH as a positive control, without altering seedling growth (Fig. 2b). ACM from strain PB69 elicited induced resistance responses against *P. syringae* pv. lachrymans (Fig. 2b). To validate defense priming, we performed qRT-PCR analysis of each plant at 0 and 3 h after pathogen challenge to measure the expression levels of the following signal marker genes: *CsLOX* (lipoxygenase) for JA signaling, *CsPR2* for SA signaling, and *CsETR* for ET signaling. *CsLOX* transcription displayed a 2-fold and 7-fold increase from 0 to 3 h post-inoculation (hpi) in SDB-treated cucumber seedlings respectively (Fig. 2c), whereas no differences in Cs*LOX* expression were observed in the BTH and control treatments (Fig. 2c). *CaPR2* transcription was upregulated 2.3-fold at 3 hpi by 1 mM BTH treatment (positive control) compared with that at 0 h, whereas the other treatments did not display significantly different expression levels at 0 and 3 h (Fig. 2d). No differences in *CsETR* expression were observed (Fig. 2e). These combined results suggest that the heat-stable metabolite from strain PB69 activates the JA-dependent signaling pathway. The strain PB69 identified as *Bacillus gaemokensis* by 16s rDNA analysis (data not shown).

### Large-scale translational field trials of SDB to cucumber plants

**Figure 3:**
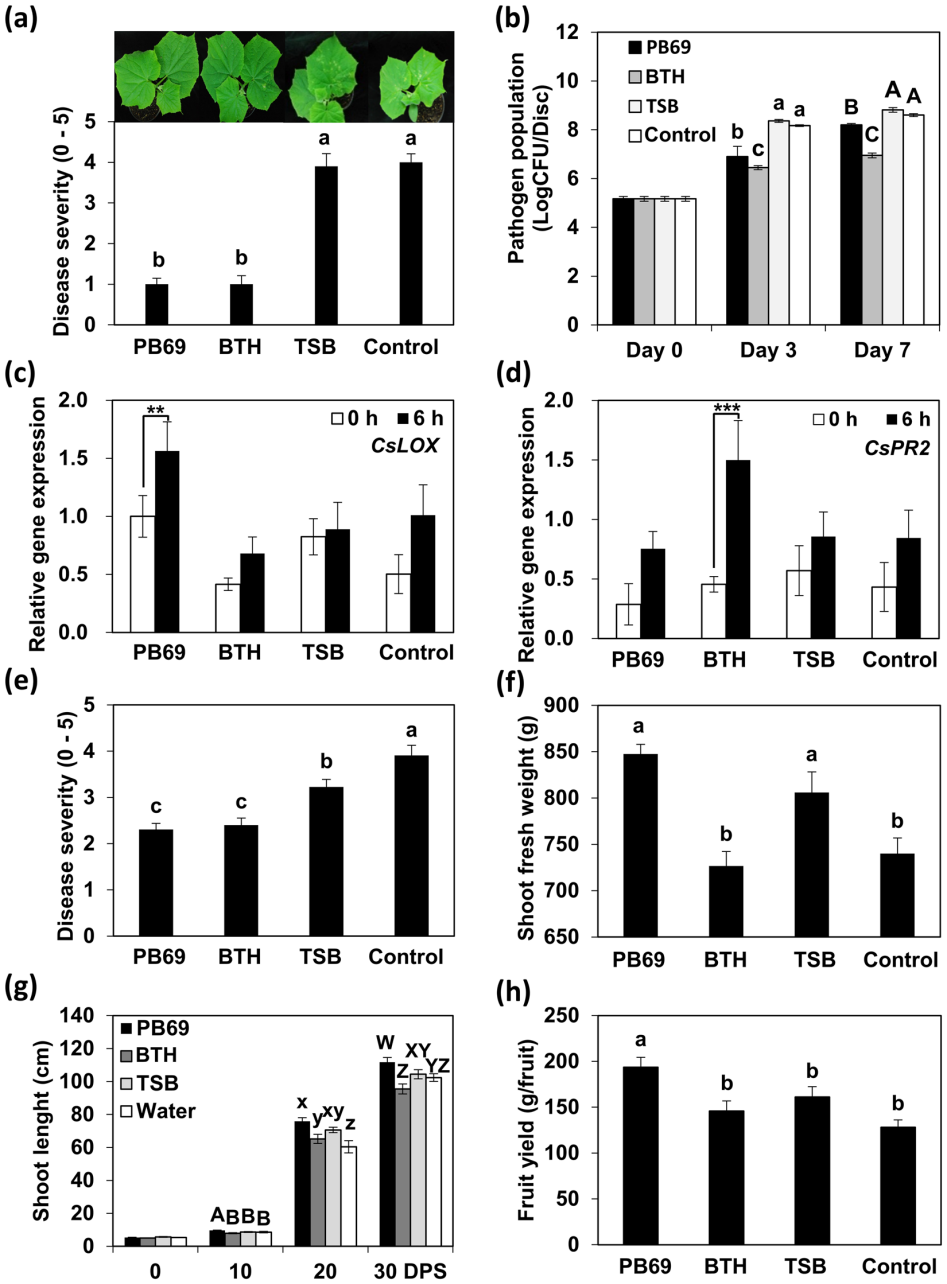
Seed defense biopriming with autoclaved culture medium from strain PB69 elicits induced resistance in cucumber plants under pot (**a**−**d**) and field conditions (**e**−**h**). Plants were kept under a 15 h day/9 h night regime at 26.8 °C (mean) for pot and field conditions. (**a**) Disease symptom severity (0–5) in SDB-treated cucumber plants was recorded at 7 days after infection with *P. syringae* pv. lachrymans (10^6^ CFU/mL) as follows: 0, no symptoms; 1, yellowish color; 2, chlorosis only; 3, partial necrosis and chlorosis; 4, necrosis of the inoculated area and expanded chlorosis; and 5, complete necrosis of the inoculated area. Photographs of the lesions were taken at 7 days after infection with *P. syringae* pv. lachrymans. (**b**) Pathogen population was measured at 0, 3, and 7 days after challenge with *P. syringae* pv. lachrymans. (**c** and **d**) Expression levels of the cucumber resistance genes *CsLOX* (two-way ANOVA, *n* = 3 experiments, **P < 0.01, F(7,16) = 1.93) and *CsPR2* (two-way ANOVA, n = 3 experiments, ***P < 0.001, F(7,16) = 4.98) were assessed by qRT-PCR analysis at 0 and 6 h after challenge with *P. syringae* pv. lachrymans. (**e**) Disease severity was measured 7 days after *P. syringae* pv. lachrymans under field conditions. (**f**) Shoot fresh weight was assessed at 50 days post-sowing (dps). (**g**) Shoot length was assessed every 10 dps during 0−30 (interval 10) dps. (**h**) Fruit yield of cucumber plants subjected to seed defense biopriming (SDB) with PB69 ACM was assessed at 50 dps. Water and 1 mM BTH were used as negative and positive controls, respectively. Different letters indicate significant differences between treatments (*P* = 0.05 according to least significant difference). Means in columns followed by asterisks are significantly different at *P* < 0.01 (**) and *P* < 0.001 (***) according to the LSD test. Error bars indicate the standard error (*n* = 16).

Translational research achieves rapid advances from basic research to large-scale applications. Therefore, we evaluated whether SDB with the heat-stable metabolite from strain PB69 elicited induced resistance against *P. syringae* pv. lachrymans under greenhouse conditions. At 28 days post-sowing (dps), SDB plants that were grown from seeds treated with PB69 ACM had disease severity of 1.0 (on a scale of 0−5), whereas the disease severity of plants primed with Tryptic soy broth (TSB, media control) or water (control) was 3.9 and 4.0, respectively (Fig. 3a). The disease severity of plants treated with 1 mM BTH (positive control) was 1.0 (Fig. 3a). We also measured *in planta* growth of *P. syringae* pv. lachrymans. At 3 and 7 days post-inoculation (dpi), the pathogen population in SDB plants from seeds treated with PB69 ACM was significantly lower than that in control plants (Fig. 3b). At 3 dpi, SDB plants had log 6.9 CFU/leaf disk, whereas the control had log 8.6 CFU/leaf disk (Fig. 3b). To confirm that PB69 metabolites elicited ISR, we performed qRT-PCR analysis to determine the expression levels of defense-related genes *CsLOX* (JA signaling) and *CsPR2* (SA signaling) after 0 and 6 h of pathogen challenge. *CsLOX* expression was higher at 6 hpi in SBD plants than in control plants (Fig. 3c). By contrast, *CsPR2* expression did not significantly differ in SBD plants and control plants (Fig. 3d), although *CaPR2* expression displayed a 3.3-fold increase from 0 to 6 hpi after seeds were treated with autoclaved BTH. These results indicate that the metabolites in PB69 ACM can induce resistance against *P. syringae* pv. lachrymans via JA-dependent signaling, even under greenhouse conditions.

Next, we evaluated whether PB69 ACM can induce plant resistance and promote growth under field conditions. Plants grown from cucumber seeds primed with PB69 ACM displayed reduced disease severity in an open field at 28 dps (i.e., 7 days after spray-challenge with *P. syringae* pv. lachrymans) (Fig. 3e). Priming cucumber seeds with PB69 ACM led to a 41% reduction in disease symptom severity compared with that of the water control (Fig. 3e). Plants grown from seeds primed with PB69 ACM showed similar levels of disease severity as those primed with BTH. To determine the effects of SDB on plant growth, we evaluated shoot length, fresh weight, and fruit yield (Fig. 3f−h). Plants grown from seeds primed with PB69 ACM produced significantly higher shoot fresh weight than those from seeds primed with BTH or water (Fig. 3f). The shoot fresh weight did not significantly differ after priming with TSB or PB69 ACM. SDB with PB69 ACM also produced higher fruit yields at harvest (50 dps) than those primed with BTH, TSB, or water (Fig. 3h). The total weight of cucumber fruit per plant was 192 g for PB69 ACM, 142 g for BTH, 157 g for TSB, and 128 g for the control (Fig. 3h). These results indicate that PB69 ACM did not inhibit plant vegetative growth, but did increase the yield of cucumber plants.

### SDB against the insect pest *Spodoptera litura*

**Figure 4:**
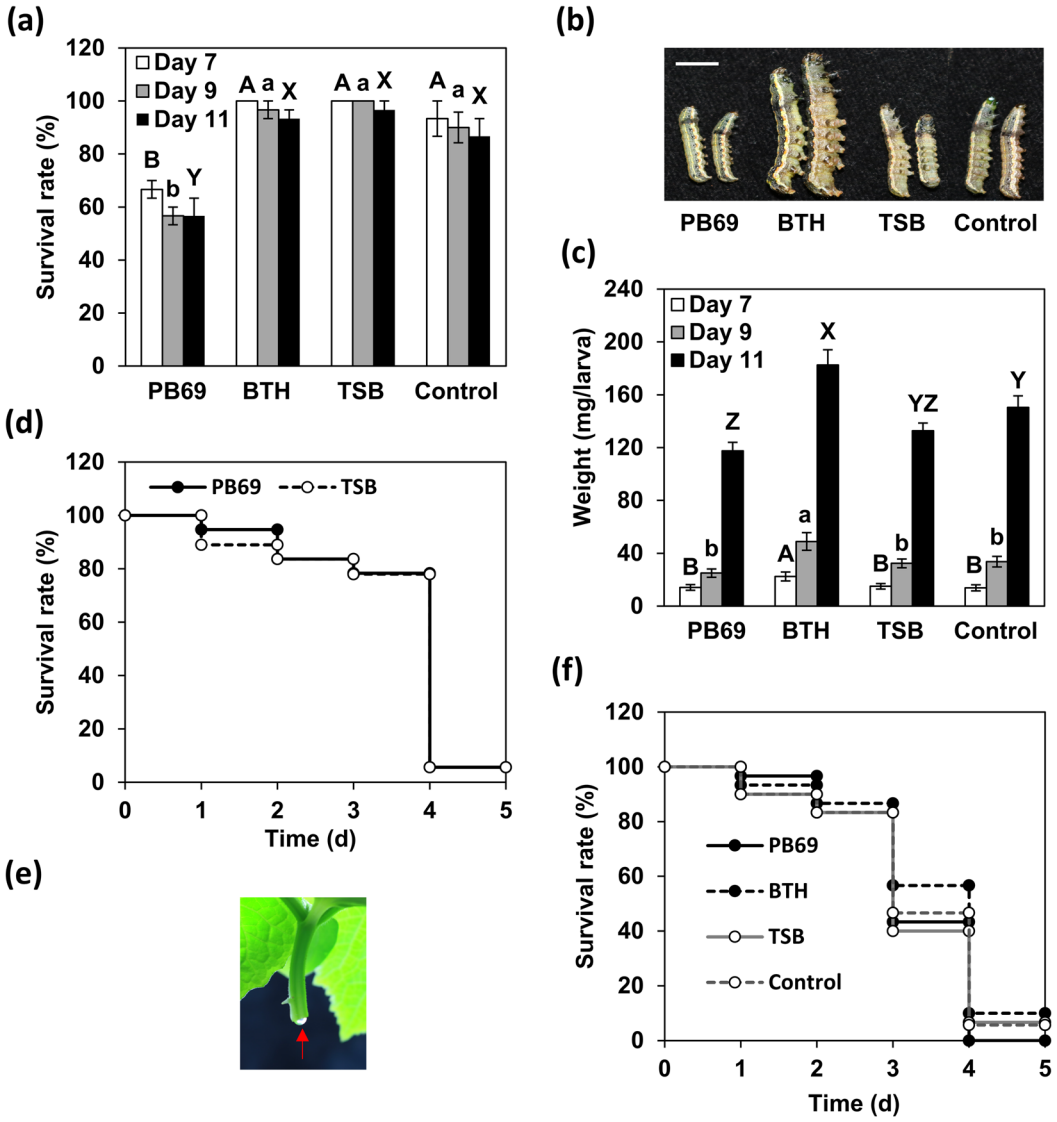
Weight and survival of *Spodoptera litura* larvae feeding on cucumber plants that were subjected to SDB with PB69 ACM. First instar larvae were evaluated for survival (%, **a**) and weight (**b**,**c**) at 7 (*n* ≥ 30), 9, and 11 days (*n* ≥ 11) after feeding on 14-day-old SDB-treated cucumber plants. Representative image at 9 days (**b**) is shown; scale represents 5 cm. (**d** and **e**) Thirty *S. litura* larvae per treatment were injected with PB69 ACM (**d**) or cucumber sap (**f**); subsequent survival was recorded daily. (**e**) Representative image of cucumber sap. Different letters indicate significant differences between treatments (P = 0.05 according to least significant difference).

To evaluate the effect of PB68 ACM on induced resistance against chewing insect herbivore, the survival and development of *Spodoptera litura* larvae (tobacco cutworm) were evaluated. The survival of *S. litura* larvae that fed on cucumber plants treated with PB69 ACM SDB was lower at 7–11 days (interval 2) after feeding than that of the controls (Fig. 4a). At 7 days, the survival of *S. litura* larvae feeding on SDB cucumber plants was 27% lower than that of the controls (93% survival) (Fig. 4a), and the survival at 9 and 11 days was similar (Fig. 4a). At 11 days, the weight of *S. litura* larvae feeding on SDB cucumber plants was 117 mg, which was 33 mg lower than that of the controls (150 mg) (Fig. 4b,c). Surprisingly, the weight of *S. litura* larvae feeding on cucumber plants grown from seeds treated with 1 mM BTH was higher than that of all other groups throughout the entire test period (Fig. 4b,c). We then assessed whether the decline in *S. litura* survival mediated by SDB with PB69 ACM was due to PB69 ACM or plant toxins. When PB69 ACM was injected into *S. litura*, survival did not differ from that of the control (Fig. 4d). Injecting the sap of SDB cucumber plants into *S. litura* also did not affect larval survival (Fig. 4f). These combined results indicate that the survival of *S. litura* larvae feeding on cucumber plants grown from seeds treated with PB69 ACM SDB was affected when they directly ate the cucumber plants.

### Pepper field application of SDB

**Figure 5:**
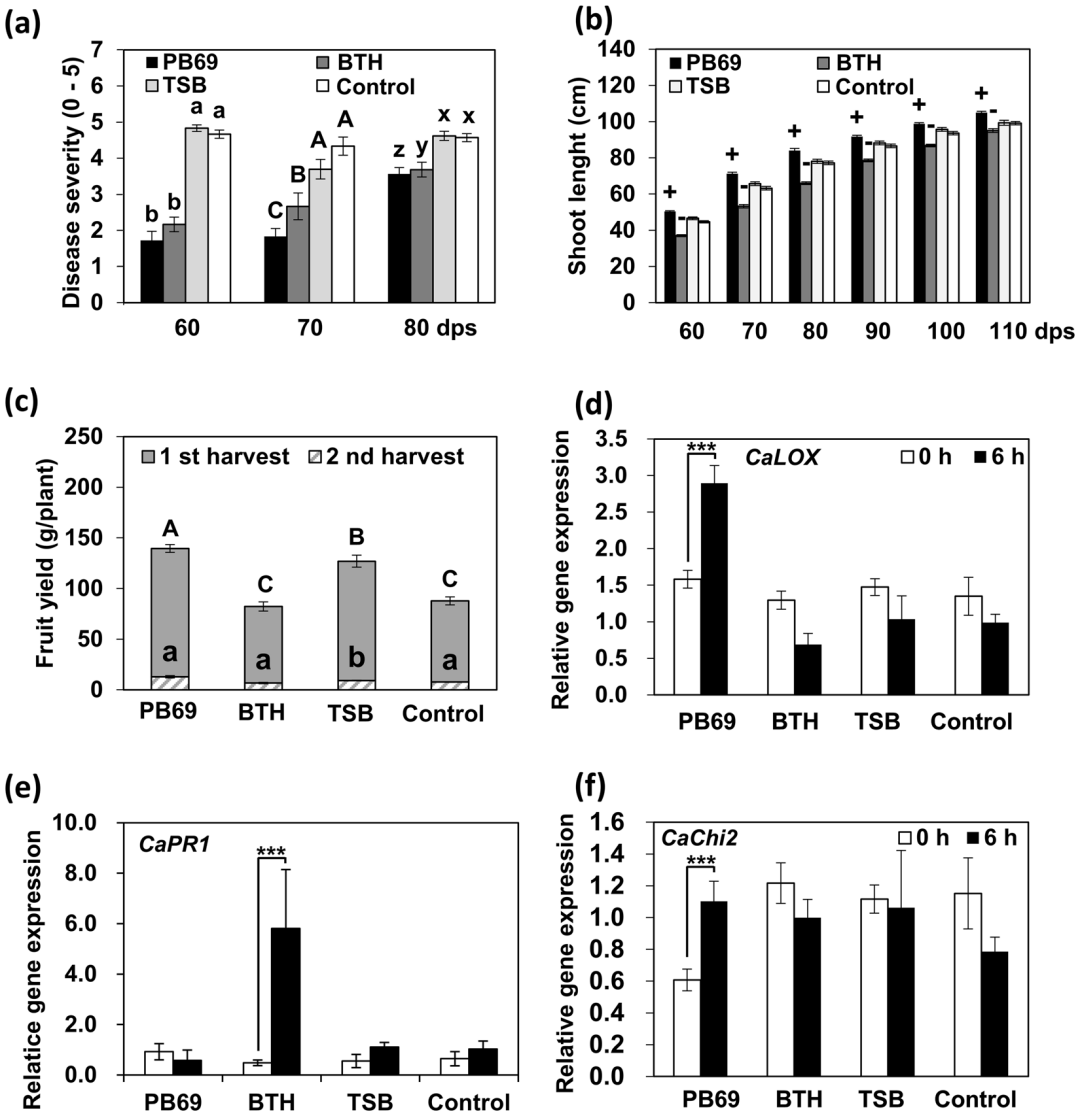
Translational approach for seed defense biopriming of pepper plants with PB69 ACM under field conditions. (**a**) Disease severity of pepper plants subjected to SDB with PB69 ACM was assessed 7 days after infection with *P. syringae* pv. lachrymans at 60, 70, and 80 dps. (**b**) Shoot length was assessed every 10 dps during 60−110 dps. (**c**) Fruit yield of pepper plants subjected to SDB with PB69 ACM was assessed at 130 and 150 dps. (**d**−**f**) Expression levels of the cucumber resistance genes *CaLOX* (two-way ANOVA, *n* = 3 experiments, ***P < 0.001, F(7,16) = 7.96) (**d**), *CaPR1* (two-way ANOVA, *n* = 3 experiments, ***P < 0.001, F(7,16) = 14.45) (**e**), and *CaChi2* (two-way ANOVA, *n* = 3 experiments, ***P < 0.001, F(7,16) = 3.67) (**f**) were assessed by qRT-PCR at 0 and 6 h after infiltrating pepper with *X. axonopodis* pv. vesicatoria. Water and 1 mM BTH were used as negative and positive controls, respectively. Different letters indicate significant differences between treatments (*P* = 0.05 according to least significant difference). An asterisk (***) indicates a significant difference (*P* < 0.001). Error bars indicate the standard error (*n* = 30).

To evaluate whether SDB is effective for other crop plants under field conditions, we tested pepper plants as a crop model. At 60, 70, and 80 dps, the disease severity in SDB pepper plants derived was 1.7, 1.8, and 3.5, respectively, whereas it was 4.6, 4.3, and 4.5, respectively, for the mock-inoculated water control (Fig. 5a). To test the effect of PB69 ACM SDB on pepper plant growth parameters, we measured shoot length and fruit yield (Fig. 5b,c). Shoot length was measured from 60 to 110 dps (interval 10), and fruit yield was assessed at 130 and 150 dps. Shoot length significantly increased in SDB pepper plants compared with the control during the entire period of shoot length measurement (Fig. 5b). Fruit yield was similar in SDB and control plants at the first harvest (130 dps), but was higher in SDB pepper plants at the second harvest (150 dps) than in plants primed with BTH, TSB, or water (150 dps) (Fig. 5c). The total weight of pepper fruit per plant was 140, 82, 126, and 88 g for plants treated with SDB, BTH, and TSB, and control plants, respectively (Fig. 5c). *CaLOX* gene expression was higher at 6 hpi in SDB plants than in control plants; expression increased 1.8-fold at 6 hpi compared with that at 0 hpi, whereas the control was downregulated 1.7-fold (Fig. 5d). *CaPR1* and *CaSAR8.2* expression did not significantly differ in SDB and control pepper plants (Fig. 5e, Supplementary Figure S2). *CaChi2* expression (marker for ET signaling) did not significantly differ between SDB pepper plants and controls (Fig. 5f). These combined results indicate that SDB with PB69 ACM enhances pepper plant vegetative growth (shoot length) and yield, and elicits induced resistance against *X. axonopodis* pv. vesicatoria under field conditions.

### Identification of the active metabolite from strain PB69

**Figure 6:**
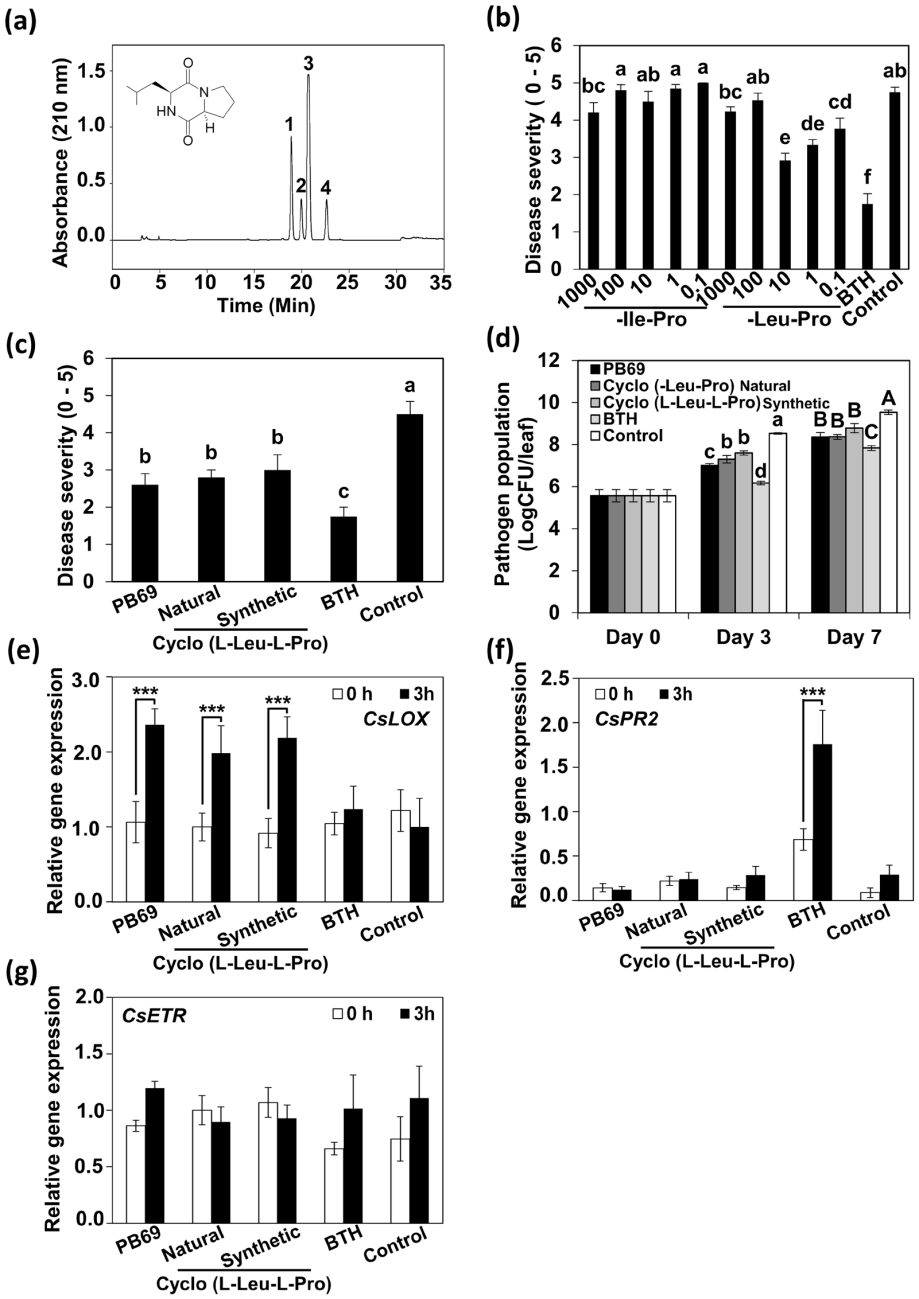
Identification of the seed defense biopriming determinant metabolites from strain PB69 ACM. (**a**) High performance liquid chromatography of compound 1−4 of strain PB69. 1, D-Ile-Pro; 2, L-Ile-Pro; 3, D-Leu-Pro; 4, L-Leu-Pro (Ile, isoleucine; Pro, proline; and Leu, leucine). (**b**) Disease severity of *in vitro* cucumber plants treated with Ile-Pro and Leu-Pro was assessed 7 days after infection with *P. syringae* pv. lachrymans. (**c**) Disease severity of *in vitro* cucumber plants treated with L-Leu-Pro was assessed 7 days after infection with *P. syringae* pv. lachrymans. (**d**) Pathogen population was measured in cucumber plants at 0, 3, and 7 days after infection with *P. syringae* pv. lachrymans. The expression levels of cucumber resistance genes *CsLOX* (two-way ANOVA, *n* = 3 experiments, ***P < 0.001, F(9,20) = 4.96) (**e**), *CsPR2* (two-way ANOVA, *n* = 3 experiments, ***P < 0.001, F(9,20) = 18.55) (**f**), and *CsETR* (two-way ANOVA, *n* = 3 experiments, F(9,20) = 2.44) (**g**) were assessed by qRT-PCR analysis at 0 and 3 h after *P. syringae* pv. lachrymans challenge. Water and 1 mM BTH were used as negative and positive controls, respectively. Different letters indicate significant differences between treatments (*P* = 0.05 according to least significant difference). An asterisk (***) indicates a significant difference (*P* < 0.001). Error bars indicate the standard error (*n* = 16).

To identify the active determinant metabolite(s) in strain PB69 that elicited ISR in cucumber and pepper plants, we conducted metabolite fractionation analysis of PB69 ACM. The fraction step was performed on a C-18 Isolute-type column. Fractionation were eluted from the silica gel with water, 50% MeOH, and 100% MeOH, and were assayed for ISR (Fig. 1). Finally, two sets of cyclodipeptides (Ile-Pro and Leu-Pro) were identified as potential active metabolite elicitors (Fig. 6a). We found the optimal concentration levels that trigger induced resistance in cucumber (Fig. 1). The Leu-Pro cyclodipeptide conferred disease resistance at 0.1–10 µM, whereas the Ile-Pro cyclodipeptide did not confer induced resistance at all concentration levels (Fig. 6b). Commercially available cyclo(L-Leu-L-Pro) conferred similar induced resistance as PB69 ACM and the fraction containing cyclo(L-Leu-L-Pro) (Fig. 6c,d). At 3 and 7 dpi, the growth of *P. syringae* pv. lachrymans was lower in SDB plants and plants treated with the cyclo(L-Leu-L-Pro) fraction and synthetic cyclo(L-Leu-L-Pro) than in the controls (Fig. 6d). At 3 dpi, the bacterial population in plants treated with the fractionated cyclo(L-Leu-L-Pro) and synthetic cyclo(L-Leu-L-Pro) was log 7.3 and 7.6 CFU/leaf disk, respectively, whereas the control was log 8.5 CFU/leaf disk (Fig. 6d). At 7 dpi, the bacterial population in plants treated with the fractionated cyclo(L-Leu-L-Pro) and synthetic cyclo(L-Leu-L-Pro) was log 8.3 and 8.7 CFU/leaf disk, respectively, whereas the control was log 9.3 CFU/leaf disk (Fig. 6d).

To confirm that treatment with fractionated cyclo(L-Leu-L-Pro) elicits induced resistance, we performed qRT-PCR analysis of defense-related gene expression of *CsLOX* (JA signaling), *CsPR2* (SA signaling), and *CsETR* (ET signaling) after 0 and 3 h of pathogen challenge. *CsLOX* expression was higher at 3 hpi after SDB with fractionated cyclo(L-Leu-L-Pro) than in control plants; a 1.9-fold and 2.4-fold increase in expression was detected in SDB plants treated with fractionated and synthetic cyclo(L-Leu-L-Pro) respectively compared with expression at 0 h, whereas the control was not significantly upregulated between 0 and 3 h (Fig. 6e). *CsPR2* and *CsETR* expression levels did not significantly differ among the tested SDB agents and control (Fig. 6f,g). *CsPR2* expression showed a 2.6-fold increase from 0 h to 3 hpi after SDB with BTH. These results indicate that cyclo(Leu-Pro) elicits induced resistance against *P. syringae* pv. lachrymans mediated by JA-dependent signaling under *in vitro* conditions.

## Discussion

This study developed a novel seed priming methodology designated as seed defense biopriming (SDB) using autoclaved rhizosphere bacterial culture metabolites, which elicit plant immunity without imposing a plant growth penalty and increase fruit yield under greenhouse and field conditions. Our results indicate that SDB was stable and provided consistent efficacy. Two cyclodipeptides (Leu-Pro and Ile-Pro) were identified as the bacterial metabolites that elicited plant resistance. Our translational research provides a new disease control strategy for important crop plants that appears robust even under field conditions.

We used cucumber as a model plant to develop a rapid *in vitro* screening method because of their faster growth, which required only 2 weeks to complete one cycle of screening. Previous studies used *Arabidopsis* as a model system; however, the resistance induced in *Arabidopsis* is not always replicated in crop plants^41^. For example, a previous study showed that a model legume and *Arabidopsis* exhibited different induced resistance responses to *Rhizoctonia solani* ^42^. SDB of *Arabidopsis* seed is technically challenging because of the small seed size and non-uniform seed germination and symptom development. By contrast, cucumber seeds are larger for experimental manipulation, the plants grow relatively quickly, and they have a well-established and consistent pathosystem (with *P. syringae* pv. lachrymans) and symptom development. Quantification of pathogen growth using GFP fluorescence intensity screening saved considerable time and effort. The use of automated systems to measure fluorescence also will reduce the screening time for potential agents eliciting disease resistance. The use of a water-agar system for screening enables large-scale screening and facilitates comparisons with *in vivo* and *in planta* experiments. The use of water-agar for cucumber seed germination allowed us to measure the effect of SDB on cucumber immunity while eliminating nutrient-mediated side effects^43^. We chose BTH as a positive control because it is a strong chemical trigger against diverse pathogens and insect pests *in vitro* and in the field. The selected ACM from strain PB69 consistently elicited ISR in repeated greenhouse and field experiments. The autoclave step eliminated possible interference from known bacterial proteins and large metabolic compounds. Previous work reported that LPs from *Bacillus* spp. and *Pseudomonas* spp. elicited ISR^20,27,44,45^. The main LPs synthesized by *Bacillus* spp. belong to the surfactin, iturin, and fengycin families^39^. These LP compounds are not normally stable to high temperature. Our approach was designed to identify new heat-stable compounds derived from *Bacillus* spp., such as the novel cyclodipeptide ISR elicitors.

PGPR-mediated ISR is distinct from SAR because ISR mostly requires JA- and ET-dependent pathways, whereas SAR requires the SA pathway. We observed JA-dependent *CsLOX* expression in SDB cucumber plants grown from seeds treated with PB69 ACM (Figs 2c and 3c). The time needed for defense-related gene expression after pathogen treatment significantly differs between cucumber grown *in vitro* (3 h) and in pot or field conditions (6 h). This discrepancy might be caused by two key differences between the groups: (1) different methods of pathogen treatment were used (dropping versus spraying, respectively); and (2) the sites of pathogen treatment differed (cotyledon versus second leaf, respectively). However, the exact reason for the different temporal responses is unknown. We also found that field-grown pepper plants required 6 h to express defense-related genes, which suggests that plant type and developmental stage also might cause discrepancies in temporal responses. Plants grown from SDB seeds treated with PB69 ACM did not grow significantly differently than control plants under greenhouse conditions, but grew faster under field conditions, which we attribute to the expression of defense-related marker genes, and reduced susceptibility to diseases compared with control plants (Figs 3g and 5d). We found that SDB seeds treated with PB69 ACM did not elicit JA signaling-dependent genes *CsLOX* (Figs 2c and 3c) in cucumber or *CaLOX* (Fig. 5f) in pepper at 0 h, but the expression of these genes was strongly upregulated at 6 h. By contrast, SAR-related genes were induced even without pathogen challenge^40^. The combined results indicate that plant defense induced by SDB with PB69 ACM is activated through typical plant defense priming pathways^40^.

These transcriptional data prompted us to evaluate the effect of PB69 ACM on induced resistance against herbivores. Previous work reported that JA signaling is involved in induced immunity against *Spodoptera* spp. larvae^46,47^. The mortality of *Spodoptera litura* larvae (tobacco cutworm) feeding on SDB cucumber plants was greater than for other treatments (Fig. 4). There are two possible explanations for this: (1) direct inhibition by PB69 ACM, although direct feeding of PB69 ACM did not kill *S. litura* larva, indicating that activation of plant defense against *S. litura* is responsible rather than direct inhibition by ACM; and (2) plant-derived metabolite that is toxic for the herbivore. Extensive research with numerous plant species has identified many small plant metabolites with toxic or antifeedant effects on insect herbivores, including terpenoids, alkaloids, furanocoumarins, cardenolides, tannins, saponins, glucosinolates, and cyanogenic glycosides^48^. The complex mixtures of toxins generated by many plants may synergistically provide defense against herbivory. For example, a combination of two monoterpenoids is approximately ten times more toxic to *S. litura* than would have been predicted from a simple additive effect^49^.

We also observed a significant increase in the expression of JA-dependent lipoxygenase (LOX) in SDB cucumber plants grown from seeds treated with PB69 ACM. LOX converts linolenate (derived from membrane lipids via lipases) to hydroperoxy derivatives, which provide a source for JA biosynthesis, including a 13-LOX that produces 13-hydroperoxy-octadecatrienoic acid, a substrate for several enzymes, including allene oxide synthase in JA biosynthesis^50^. Oxidative enzymes such as LOX covalently modify dietary protein through the production of reactive o-quinones and lipid peroxides^51,52^. Catalysis by O_2_-dependent enzymes is limited by low oxygen levels in the foregut and midgut of some insect species^53^, so an alternative possibility is that *LOX* expression rapidly changes in response to tissue mastication by insects. The mortality of larvae that ate PB69 ACM-treated cucumber plants was higher than the control. Zebelo *et al*. (2016) reported similar results, in which *Spodoptera exigua* showed lower growth on PGPR-treated cucumber plants^54^. However, we used autoclaved conditioned medium without bacterial cells to eliminate possible effects of known compounds. *S. litura* weight also increased from feeding on plants grown from BTH-treated seeds. *LOX* gene expression was the lowest in BTH-treated plants, because BTH strongly induces SA signaling, which is antagonistic to JA signaling (Fig. 3c,d). SA considerably affects JA-dependent signaling^9,55,56^. For example, silver leaf whiteflies use SA-JA crosstalk to activate the SA pathway and consequently suppress JA-mediated defense, which accelerates their development^57^.

*Bacillus gaemokensis* strain PB69 elicited induced resistance in cucumber and pepper, enhanced fruit yield in pepper, and protected plants against bacterial pathogen infection (Fig. 5c). Several studies reported negative effects of BTH, SA, and BABA on plant growth and yield in the absence of pathogen pressure^58–60^, as direct induction of defense responses is likely to be wasteful in the absence of disease. By contrast, defense priming activates defense responses to pathogen challenge. We found that crop yield did not decline in pepper plants grown from SDB seeds with PB69 ACM compared with control plants. By contrast, BTH-treated cucumbers displayed considerable growth inhibition but similar yield as control plants. Therefore, SDB appears to have clear ecological benefits and represents a promising approach for crop protection. Many studies have tested whether these agents are effective at defending against diseases in the field, but it is not easy to determine what amounts are needed to have field effects. Occasionally, disease resistance disappears after interacting with other ecosystems. To achieve more successful translational field applications of these agents, a greater understanding of their ecology is needed. During laboratory screening, we found that certain agents of a single bacterium induced disease resistance. During a field test, we found that these agents showed stable results in cucumber. We then applied it to pepper, and observed induced disease resistance in the field. This represents a successful plant translational study, which can be used to enhance agricultural productivity. We also observed that TSB treatment showed positive effects on growth and induced resistance in some cases (Figs 3c,f, 5c). A possible explanation is that TSB contains tryptone, phytone, NaCl, dipotassium phosphate, and glucose, which may elicit plant growth and induced resistance. However, the effectiveness of TSB was not consistent in different experiments (data not shown). Future work will perform a detailed investigation of specific determinants in TSB.

We identified two cyclodipeptides [cyclo(Ile-Pro) and cyclo(Leu-Pro)] that induced disease resistance in cucumber and pepper (Fig. 6a). Cyclic dipeptides are among the most common peptide derivatives found in nature, and they exhibit many biological functions, including antibacterial, antifungal, antiviral, antitumor, and antitoxin activities^61,62^. Among the many cyclic dipeptides isolated from nature, cyclo(L-Leu-L-Pro) has been isolated from many organisms, including *Streptomyces*, *Rosellinia necatrix*, *Rhaphisia pallida* (marine sponge), and *Halobacillus litoralis* (marine bacterium). It has been reported that cyclo (Pro-Leu) retards the growth of rice seedlings and roots, and that it is effective against vancomycin-resistant enterococci and leukemic cells^61^. The induced resistance activity of cyclo dipeptides depends on its peptide structure, because cyclo(Ile-Pro) did not show the same induced resistance against *P. syringae* pv. lachrymans in cucumber [at 100 and 0.01 mM cyclo (L-Leu-L-Pro)]. A single amino acid or a combination of two amino acids did not result in any pathogen inhibition at the concentrations investigated^61^. The cyclodipeptide inhibitory activity appeared to crucially depend on the presence of proline and a hydrophobic amino acid. Other cyclodipeptides without proline did not show the same activity. The requirement for leucine or valine suggests that a hydrophobic interaction may be involved in the activity.

**Figure 7:**
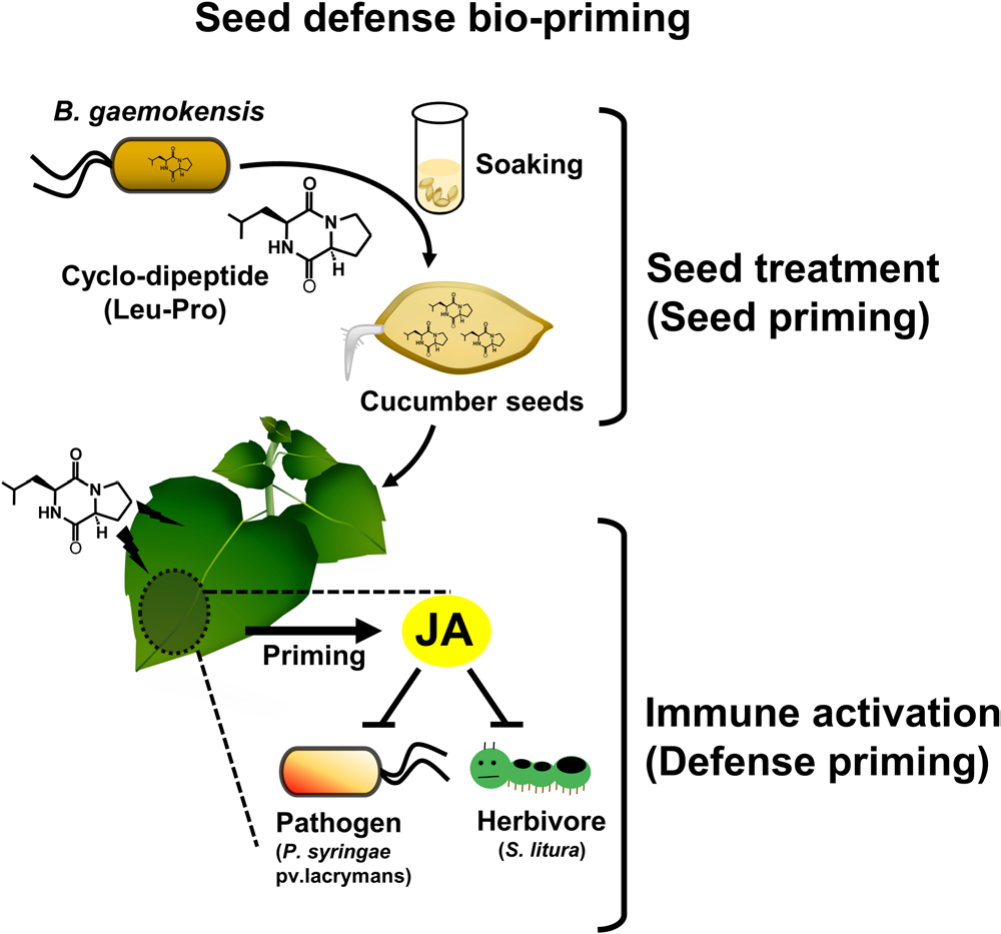
A model depicting the mechanism of seed defense biopriming with PB69 ACM.

In conclusion, we demonstrate for the first time that cyclo(L-Leu-L-Pro) is effective as a SDB agent, and can be used to induce plant resistance against *P. syringae* pv. lachrymans in cucumber and against *X. axonopodis* pv. vesicatoria in pepper (Fig. 7). SDB with cyclo(L-Leu-L-Pro) is a promising technique for protecting plants against pathogens and insect pests by enhancing plant defenses in the field.

## Methods

### Plant growth conditions for optimizing cucumber seed biopriming

Cucumber seeds (*Cucumis sativus* L. cv. Backdadagi) were surface-sterilized with 3% sodium hypochlorite for 5 min, and then rinsed five times with sterile distilled water (SDW). Then, seeds were submerged at room temperature in 1 mM BTH for 0, 6, 12, and 18 h. Primed seeds were dried on filter paper for 12 h. The seeds were then planted in autoclaved soil for pot experiments, or in water-agar medium for *in vitro* experiments. Bacterial pathogens were cultured overnight at 28 °C in King’s B medium supplemented with 100 μg mL^−1^ rifampicin. A culture of the compatible bacterial pathogen *Pseudomonas syringae* pv. lachrymans (suspended in 10 mM MgCl_2_ with OD_600_ = 1) was sprayed until run-off (potted plants) or inoculated (*in vitro* plants) on cucumber seedling leaves at 14 and 7 days after planting, respectively. Plants were returned to the growth chamber immediately after inoculation. Induced resistance to *P. syringae* pv. lachrymans*::*green fluorescence protein (GFP) was evaluated after 28 days for potted plants and after 14 days for *in vitro* plants. Disease symptom severity was scored from 0 to 5 as follows: 0, no symptoms; 1, less than 20% diseased area; 2, 21–40% diseased area; 3, 41–60% diseased area; 4, 61–80% diseased area; and 5, more than 81% of the whole leaf was diseased. Leaves were harvested at 0, 3, and 6 h after inoculation and immediately frozen in liquid nitrogen for total RNA extraction. Intact cucumber leaves were used for non-stress treatments.

### Preparation of autoclaved bacterial culture from strain PB69

The identity of *Bacillus gaemokensis* strain PB69 was verified by PCR amplification of the 16 S rRNA gene, and then the 16 S rRNA gene sequence was compared with other sequences in GenBank. This strain is deposited in the Korean collection for type cultures, KRIBB, Republic of Korea (KCTC12299BP). The PB69 strain was cultivated in TSB in the dark for 72 h at 30 °C using a rotary shaker at 200 rpm. The PB69 culture was autoclaved, and this preparation was used to identify heat-stable compounds that could elicit induced resistance in cucumber plants.

### Plant preparation for *in vitro* and field experiments

Cucumber seeds were submerged for 12 h in SDW containing 1 mM BTH, and then seeds were air-dried on filter paper. Primed seeds were planted in water-agar for *in vitro* experiments and in soil-less medium in pots (Punong Co. Ltd, Gyeongju, Korea) under greenhouse conditions at 28 °C under a 16 h light/8 h dark photocycle for field experiments. Disease assays and plant sampling were conducted as described previously^23^. Primed cucumber plants were cultivated in an open field under natural conditions. For greenhouse experiments, seeds were planted in a 24-hole plug tray containing soil (Punong Co. Ltd, Gyeongju, Korea), and plants were grown under greenhouse conditions. Then, seedlings were transplanted into large pots (d = 30 cm and height = 30 cm). For positive and negative controls, plants were treated with 0.5 mM BTH or water, respectively.

Pepper seeds were submerged in SDW containing 1 mM BTH for 36 h, and then seeds were air-dried on filter paper. Primed pepper plants (*Capsicum annuum* L. cv. Bukwang) were cultivated in a growth chamber under a 16 h light/8 h dark photocycle at 25 °C. Pepper seedlings were grown in a growth chamber in the dark at 25–28 °C. Germinated pepper seeds were transferred to sterilized soil containing a nutrient soil-less mixture (Punong Co. Ltd, Gyeongju, Korea), and plants were cultivated in the greenhouse for 3 weeks. Induced resistance was tested under field conditions by transplanting pepper seedlings at a distance of 40 cm apart in the field. Before transplanting seedlings, each row was covered with black and white polyethylene plastic film. Pepper seedlings were grown in beds that were 20 cm high, 30 cm wide, and 880 cm long. Single-row plots with 30 plants per plot were replicated four times in a completely randomized design.

Plants were challenged with pathogen as follows. A culture of the compatible bacterial pathogen *Xanthomonas axonopodis* pv. vesicatoria (OD_600_ = 0.04 in 10 mM MgCl_2_) was pressure-infiltrated into the abaxial surface of pepper leaves 3 weeks after transplant using a needleless syringe. Seven days after pathogen challenge, disease severity was assessed as described previously^22^. Bacterial pathogens were cultured overnight at 28 °C in LB medium supplemented with the appropriate antibiotics. Leaves were harvested at the indicated times and then immediately frozen in liquid nitrogen for total RNA extraction. Intact pepper leaves were used for non-stress treatments. After pathogen inoculation with *X. axonopodis* pv. vesicatoria, plants were returned to the growth chamber and leaf tissue was harvested at 0, 3, and 6 h after inoculation, and used for isolation of total RNA for gene expression.

### Induced resistance against insect pest

*Spodoptera litura* eggs were purchased from Farm Hannong Research (Seoul, Korea). After hatching, neonate larvae were transferred to feed on artificial media for 2 days. An acclimation period is required whenever larvae are transferred from one diet to another; therefore, 1 day before the experiment, second-instar larvae were transferred to feed on non-experimental cucumber plants. After this pre-feeding, larvae of the same developmental stage were weighed and transferred to 14-day-old SDB-treated cucumber plants. Larval weight and survival were recorded after 7, 9, and 11 days of feeding. Sap from 14-day-old SDB-treated cucumbers (20 μL) was injected into the hemolymph of ultimate‐instar *Spodoptera litura* larvae using a 5 μL microsyringe (Microliter TM #701, Hamilton). Ten larvae were injected per treatment and kept in Petri dishes at 28 °C in the dark, and the experiments were repeated three times. Larvae were regularly scored as alive or dead during 3 days.

### Measurement of plant growth parameters

We tested whether seeds primed with PB69 have reduced growth and yield under field conditions by measuring plant height every 10 days from 10 to 60 dps and measuring the total plant yield (total fruit weight per treatment per plant) at 64 and 77 dps. This experiment was repeated four times.

### RT-PCR and quantitative RT-PCR analyses

Total RNA was isolated from cucumber and pepper leaf tissues using Tri Reagent (MRC, Cincinnati, OH, USA) according to the manufacturer’s instructions. RT-PCR (RETROscript; Ambion) analysis was performed according to the manufacturer’s instructions. RT-PCR and quantitative RT-PCR (qRT-PCR) were conducted as described previously^29^. *PR-2*, *LOX*, and *ETR* gene expression levels were determined in cucumber using the primers shown in Table 1. *PR-1*, *PR-4*, *LOX*, *Chi*, and *SAR8.2* gene expression levels were determined in pepper using the primers shown in Table 1. The qRT-PCR reactions were performed using the Chromo4^™^ Multicolor Real-Time PCR Detection System (Bio-Rad Laboratories, Hercules, CA, USA). The qRT-PCR reactions contained 10 μL of iQ^™^ SYBR^®^ Green SuperMix (Bio-Rad Laboratories), 3 μL of diluted cDNA, 10 pmol of each primer, and sufficient SDW to bring the final volume to 20 μL. The thermocycler parameters were as follows: initial polymerase activation for 10 min at 95 °C, followed by 40 cycles of 30 s at 95 °C, 60 s at 55 °C, and 30 s at 72 °C. Relative RNA levels were calibrated and normalized against expression levels of *CsActin* and *CaActin* mRNA (GenBank accession no. AB010922 and AY572427).

**Table 1:** List of primers forward primer (FP) and reverse primer (RP) used for amplification using qRT-PCR.

Plant	Gene Name	Primer
Cucumber	*CsPR-2*	FP: 5′-TCAATTATCAAAACTTGTTCGATGC-3′ RP: 5′-AACCGGTCTCGGATACAACAAC-3′
	*CsLOX*	FP: 5′-AAGGTTTGCCTGTCCCAAGA-3′ RP: 5′-TGAGTACTGGATTAACTCCAGCCAA-3′
	*CsETR*	FP: 5′-GCCATTGTTGCAAAAGCAGA-3′ RP: 5′-GCCAAAGACCACTGCCACA-3′
	*CsActin*	FP: 5′-CCGTTCTGTCCCTCTACGCTAGTG-3′ RP: 5′-GGAACTGCTCTTTGCAGTCTCGAG-3′
Pepper	*CaPR-1*	FP: 5′-ACTTGCAATTATGATCCACC-3′ RP: 5′-ACTCCAGTTACTGCACCATT-3′
	*CaPR-4*	FP: 5′-AACTGGGATTGAGAACTGCCAGC-3′ RP: 5′-ATCCAAGGTACATATAGAGCTTCC-3′
	*CaLOX*	FP: 5′-TGCAGGTTACCTCCCAAATCGCCCA-3′ RP: 5′-CTATATCGACACACTGTTGGGTATTCCTT-3′
	*CaChi*	FP: 5′-ATATTCCGAATGTCTAAAGTGGTAC-3′ RP: 5′-ATTGGACGATGGAAGCCATCACCAG-3′
	*CaSAR8.2*	FP: 5′-TAGTGAGACTAAGAAAGTTGGACG-3′ RP: 5′-AAGAGTGCATGCAGTATCACAAAG-3′
	*CaActin*	FP: 5′-CACTGAAGCACCCTTGAACCC-3′ RP: 5′-GAGACAACACCGCCTGAATAGC-3′

### Purification and identification of the biopriming elicitor

An ethyl acetate extract of the ACMs of the PB69 strain was subjected to silica gel column chromatography. Several fractions were collected, and these fractions were screened by conducting *in vitro* tests (Fig. 1). Then, the bioactivity of the pure compound was confirmed by testing against *P. syringae* pv. lachrymans infection. The pure compound was subjected to UV spectroscopy, nuclear magnetic resonance (NMR), and high-resolution mass spectroscopy (HRMS) analyses. The structure of the compound corresponded to a diketopiperazine (DKP), which was identified as cyclo(L-Pro-D-Leu) [(3 R,8aS)-3-(2-methylpropyl)hexahydropyrrolo[1,2-a]pyrazine-1,4-dione].

### Data analysis

Data were evaluated by computing analysis of variance (ANOVA) using JMP software v. 4.0 (SAS Institute Inc., Cary, NC, USA). The significance of direct and indirect biological and chemical treatments was determined by the magnitude of the *F* value at *P* = 0.05. When a significant *F* value was obtained for treatments, separation of means was accomplished using Fisher’s protected least significant difference (LSD) at *P* = 0.05. Statistical significance for treatment effects was determined by one-way ANOVA using the multcomp package in R version 3.4.0^63,64^. The results of repeated trials of each experiment were similar. Therefore, one representative trial of each experiment was reported.

## Electronic supplementary material

Supplementary Information
