# Supplementary material for: Seed defense biopriming with bacterial cyclodipeptides triggers immunity in cucumber and pepper
Source: Sci Rep. 2017 Oct 27;7:14209. doi: 10.1038/s41598-017-14155-9 (PMC5660235; doi:10.1038/s41598-017-14155-9)
Supplement: Supplementary file 1 — Supplementary Information [file 41598_2017_14155_MOESM1_ESM.pdf]

Supplementary information

Seed defense biopriming with bacterial cyclodipeptide triggers immunity and yield increase in cucumber and pepper

Geun Cheol Song[1], Hye Kyung Choi[1], Young Sook Kim[3], Jung Sup Choi[3], & Choong-Min Ryu[1,2,*]

[1]Molecular Phytobacteriology Laboratory, KRIBB, Daejeon 34141, S. Korea; [2]Biosystems and Bioengineering Program, University of Science and Technology, Daejeon, 34113, S. Korea; [3]Eco-Friendly New Materials Research Center, KRICT, Daejeon, 34114, S. Korea

[*] Author for correspondence:
Choong-Min Ryu
TEL: +82-+42-879-8229
FAX: +82-+42-860-4488
E-mail: cmryu@kribb.re.kr

Supplementary information

Supplementary Figure S1. Screen to identify optimal parameters for a novel seed defense biopriming protocol in cucumber.

Supplementary Figure S2. Expression levels of cucumber resistance gene *CaSAR8.2* was assessed by qRT-PCR analysis at 0 and 6 h after *X. axonopodis* pv. vesicatoria challenge.

Fig. S1

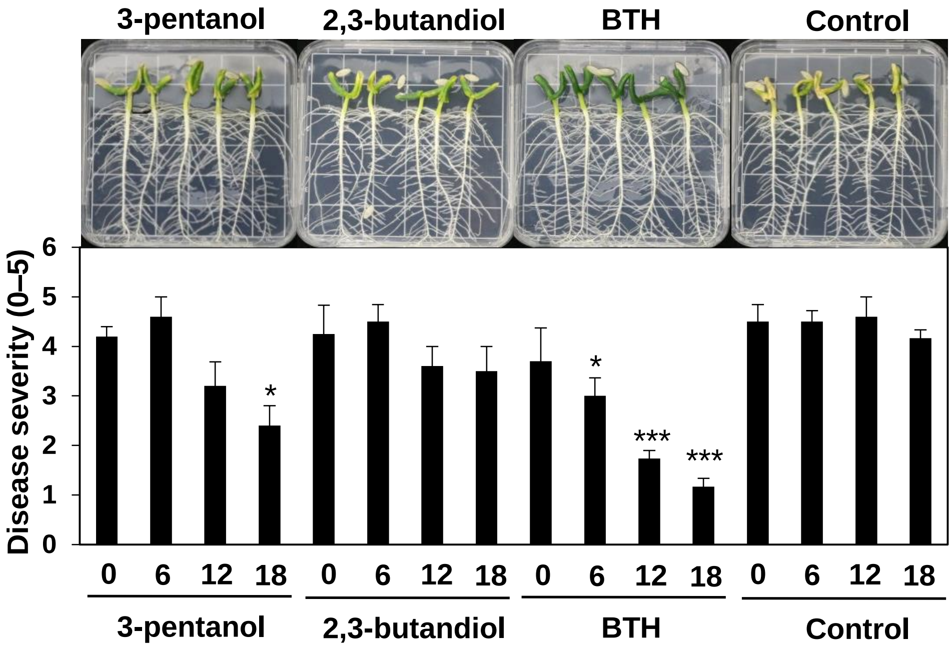
3-pentanol
2,3-butandiol
BTH
Control
Disease severity (0–5)
6
5
4
3
2
1
0
*
*
***
***
0 6 12 18
0 6 12 18
0 6 12 18
0 6 12 18
3-pentanol
2,3-butandiol
BTH
Control

Fig. S2

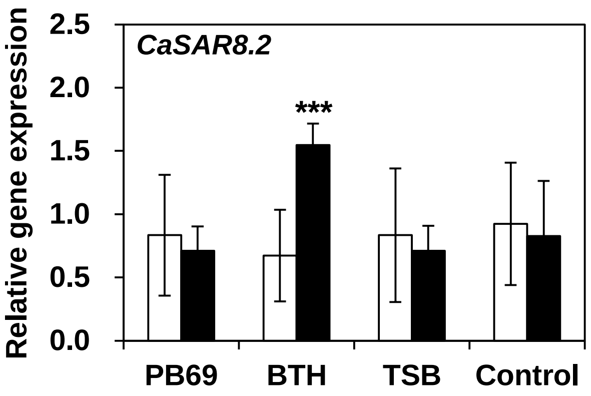

CaSAR8.2
Relative gene expression
2.5
2.0
1.5
1.0
0.5
0.0
***
PB69
BTH
TSB
Control
